# False Reassurance From a Stool Test: A Delayed Cancer Diagnosis

**DOI:** 10.7759/cureus.38107

**Published:** 2023-04-25

**Authors:** Mark S Obri, Ryan Goleniak, Mohamed Ramzi Almajed, Daniel Eid, Abigail Entz

**Affiliations:** 1 Internal Medicine, Henry Ford Health System, Detroit, USA; 2 College of Medicine, Northeast Ohio Medical University, Detroit, USA

**Keywords:** primary care, stool tests, colonoscopy, screening, colorectal cancer

## Abstract

Colorectal cancer (CRC) is an increasingly prevalent condition in the United States and the world. Numerous screening tools have been created to help prevent and identify early cases of CRC, which have led to better outcomes for patients. These screening tools range from stool tests to more invasive procedures like a colonoscopy. With this array of screening options, patients are often presented with a bevy of choices in their primary care clinic and may have difficulty understanding the difference between screening and treatment. Popular culture has also influenced these decisions as both traditional media and social media have weighed in on the experience with these screening tools. We present a unique case where our patient tested negative on a stool screening exam and proceeded to be diagnosed with CRC within the negative screening period. The case was complicated by the patient's reluctance to undergo a colonoscopy and a unique combination of symptoms that led to a difficult diagnosis.

## Introduction

Colorectal cancer (CRC) is the third most prevalent cancer worldwide; its prevalence has been increasing globally and in the United States over the past several decades. This increase in prevalence and incidence is secondary to better identification as well as increased risk factors including an aging population and obesity [1]. The advent of screening tests has resulted in earlier diagnosis of CRC, which has improved outcomes with patients experiencing decreased morbidity and mortality [2,3]. Originally, endoscopic modes of screening including colonoscopy and flexible sigmoidoscopy were the mainstay; however, newer stool-based tests have offered a less-invasive mode of screening.

Current guidelines for CRC screening published by the American Cancer Society (ACS) and the United States Preventative Services Task Force (USPSTF) offer various modalities for colon cancer screening. No recommendation is provided regarding the modality of choice for average-risk patients, which leaves the decision to the clinician and patient to arrive at the most appropriate option for each individual patient while taking comorbidities and socioeconomic factors into account. Of note, patients with an increased family risk of colon cancer or a history of inflammatory bowel disease will need colonoscopy rather than stool screening antigen and will usually have the consultation of a gastroenterologist [4]. Patients are often swayed toward less-invasive options as opposed to endoscopic screening due to concerns regarding bowel preparation, time constraints, and sedation or anesthesia. Stool-based tests are appealing as they appear less burdensome, less invasive, and less time-consuming [4]. Advertising for stool-based testing online, on television, and in popular culture also influence patients’ decisions.

Stool-based screening tests for colorectal cancer include high-sensitivity guaiac fecal occult blood test (gFOBT), fecal immunochemical test (FIT), and stool DNA test with fecal immunochemical test (sDNA-FIT). The frequency of screening stool tests varies based on the modality; high-sensitivity gFOBT and FIT are repeated yearly, whereas sDNA-FIT is repeated every three years. All three modalities have demonstrated adequate sensitivity and specificity as screening tests across a population. High-sensitivity gFOBT has a sensitivity of 50%-75% and a specificity of 96%-98% [5-8]. FIT has a sensitivity of 74% and a specificity of 94% [3]. sDNA-FIT has a sensitivity of 93% and a specificity of 84% [5-8]. A correct interpretation of these measures of screening tests and the discussion of these numbers with patients is crucial as misinterpretation poses risks of inappropriate reassurance and inadequate follow-up. Emphasis should be placed on the need for further investigations including endoscopic evaluation with direct visualization in patients with a positive screening stool-based test to arrive at a diagnosis.

Endoscopic screening tests for CRC including colonoscopy and flexible sigmoidoscopy are believed to have higher sensitivity and specificity compared to stool-based screening tests, although studies have had difficulty quantifying these parameters due to the significant heterogeneity in patient selection and operator skills. Misconceptions about colonoscopy in the media and among the community make it difficult for clinicians to discuss and recommend it as a screening test. For instance, a recent article by CNN was titled “Colonoscopies: new study questions their effectiveness” in response to a large randomized clinical trial that was performed in a small European population, which does not make it generalizable [9,10]. High-profile cases of anesthesia complications also contribute to patient hesitancy.

We present a case of a patient who had a negative sDNA-FIT test and soon thereafter developed iron-deficiency anemia and gastrointestinal symptoms but declined endoscopic testing, which delayed the diagnosis of colorectal cancer.

## Case presentation

The patient is a 72-year-old male with a notable history of hypertension, untreated hepatitis C, ischemic stroke, and tobacco abuse of one pack per day for 35 years. He initially presented to his primary care clinic to re-establish care after not being seen for years. His care was being coordinated by a family member, who had been his primary caregiver since his previous cerebrovascular accident (CVA). At that time, a DNA FIT was ordered for screening due to the patient’s lack of colon cancer screening and labs demonstrating iron-deficiency anemia. He had no family history of colon cancer and was deemed to be of average risk. He had never previously had a colonoscopy due to his preference and poor follow-up. The patient had refused colonoscopy but agreed to the DNA FIT, which was negative.

Starting approximately two months after negative mt-sDNA, the patient had multiple episodes of melanotic stools and coffee-ground emesis requiring blood transfusion and hospital admissions. Initial esophagogastroduodenoscopy (EGD) showed a single 6-mm angioectasia with successful ablation. The patient had subsequent admissions and bleeding with multiple EGDs, including with push enteroscopy, but no source was ever found. A capsule study that was said to be with poor colonic prep also only showed non-bleeding angiodysplasia lesions. Throughout these episodes, the patient initially declined colonoscopy due to comfort level. The patient eventually agreed, but the colonoscopy was deferred to an outpatient setting due to the concern that this would be an upper gastrointestinal tract bleed.

Patient imaging throughout these episodes, including computerized tomography (CT) scan of the abdomen and magnetic resonance imaging (MRI), was significant only for mildly enlarged non-specific abdominal lymph nodes that were assumed to originate from his active hepatitis C.

**Figure 1:**
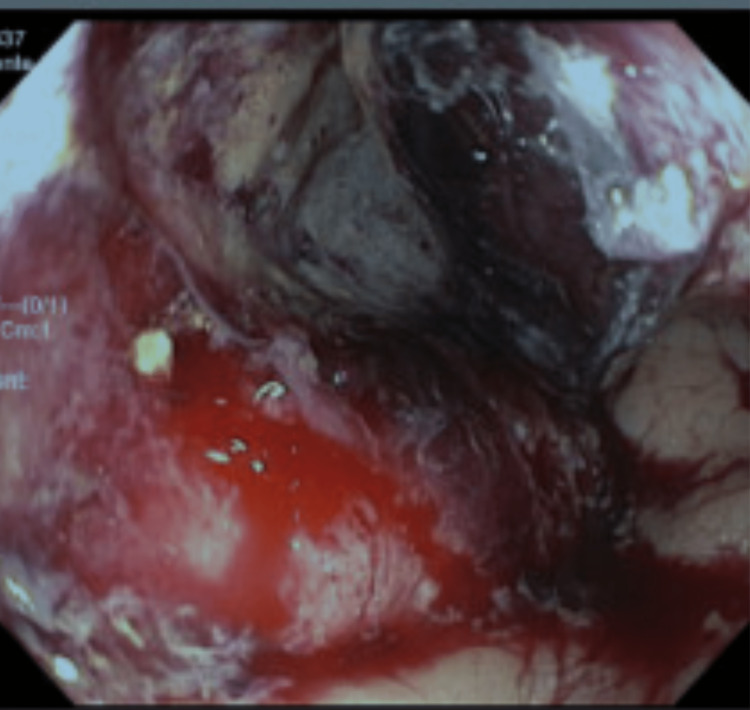
Mass seen during colonoscopy in the rectum

Eventually, the patient’s aspirin was stopped due to concerns about these recurrent bleeds with a resolution of his bleeding and no further admission. Approximately nine months after his initial episode of melena and 10 months after negative colon cancer screening, the patient agreed to have an outpatient colonoscopy for this history of melanotic stools with no definitive etiology. He was found to have an obstructing and ulcerated mass in the rectum, extending from 10 to 15 cm from the anal verge (Figure 1). Interestingly, the patient had no symptoms. Subsequent biopsy revealed invasive adenocarcinoma.

## Discussion

In this case, we had a patient with evidence of gastrointestinal (GI) bleeding and a multifactorial delay in colonoscopy in the setting of a negative mt-sDNA, which likely provided some reassurance from a CRC perspective. According to the patient and the lab, the stool test was sufficient and had no errors when used for screening, although it should be noted that user errors can often occur with the stool test. The patient ultimately received a diagnosis of CRC, which may or may not have been contributing to his bleeding. This case is not a commentary on the utility of stool screening exams but rather a cautionary tale that it is important to emphasize to patients that they may remain at risk and require physician follow-up if they have alarming signs and symptoms. Interestingly, despite having numerous EGDs, a capsule study, and a push enteroscopy with potentially an explanation for his bleeding, the patient in this case was still recommended to have a colonoscopy for definitive testing. Theoretically, a flexible sigmoidoscopy or colonoscopy would have revealed the mass sooner, but the primary team was having incredible difficulty with consenting the patient to a lower GI procedure. The patient was reassured by his stool cancer screening, although the primary team had pointed out that the presentations could also be from a lower GI bleed. The combination of stool screening, unique presentation of symptoms, and numerous upper GI procedures likely amplified the patient's thoughts that a colonoscopy was unnecessary.

CRC screening remains difficult in the United States, with a study revealing that only around 65% of the population is up to date on CRC screening [11]. Although stool antigen tests play a vital role in this screening, physicians must still explain the difference between screening in an average, healthy patient and diagnostic tests in patients with active symptoms. As described above, these tests are for a specific population, which is generally someone with no symptoms and an average colorectal cancer risk.

When interpreting screening tests, it is crucial for clinicians to discuss test characteristics with patients and explain how they apply to an individual patient. Although we refer to sensitivity and specificity to evaluate screening tests on a population level, predictive values should be used when applying the test to an individual patient. Positive predictive value (PPV) describes the probability that a patient with a positive screening test has the disease. Negative predictive value (NPV) describes the probability that a patient with a negative screening test does not have the disease. sDNA-FIT tests have a false negative rate of 7.7% and an NPV of 99.9% for the identification of CRC [12]. Clinicians should consider and communicate these statistics with patients; patients with a high suspicion of CRC should be strongly recommended for further diagnostic testing.

## Conclusions

Screening stool studies are an important tool for CRC. They offer cheaper and more convenient alternatives for screening and may convince a patient who would not have wanted screening otherwise. However, it is important to explain to patients that when presented with bleeding and alarm symptoms, it is important to undergo further testing.

In this case, we present a unique presentation that was thought to be an upper GI bleed and ended up being colorectal cancer. The combination of multiple upper GI exams and the patient's refusal to undergo colonoscopy due to his potential misunderstanding of the stool screening tests led to a likely delayed cancer diagnosis. Further education and clarification with patients are needed in the future to prevent misunderstandings about what a screening test is indicated for.
